# Comparison of the duration of viral RNA shedding and anti-SARS-CoV-2 spike IgG and IgM antibody titers in COVID-19 patients who were vaccinated with inactivated vaccines or not: a retrospective study

**DOI:** 10.1186/s12879-022-07808-2

**Published:** 2022-11-09

**Authors:** Chuancai Xu, Zhisong He, Wei Lei, Li Chen, Dan Shen, Xiaofei Wang, Deyu Xu, Ying Xu, Jian’an Huang

**Affiliations:** 1 Department of Pulmonary and Critical Care Medicine, The First Affiliated Hospital of Soochow University, NO. 899, Pinghai Street, Suzhou, 215006 China; 2 Department of Cardiology, The First Affiliated Hospital of Soochow University, NO. 899, Pinghai Street, Suzhou, 215006 China; 3 Department of Infectious Diseases, The First Affiliated Hospital of Soochow University, NO. 899, Pinghai Street, Suzhou, 215006 China; 4 Department of Nephrology, The First Affiliated Hospital of Soochow University, NO. 899, Pinghai Street, Suzhou, 215006 China

**Keywords:** SARS-CoV-2, Breakthrough infection, Inactivated vaccine, Viral RNA shedding, SARS-CoV-2-specific IgG, SARS-CoV-2-specific IgM

## Abstract

**Background:**

At present, the role of inactivated vaccines in viral RNA shedding among Severe Acute Respiratory Syndrome Coronavirus 2 (SARS-CoV-2) breakthrough infections is still unknown.

**Methods:**

We collected data of 147 coronavirus disease 2019 (COVID-19) patients with mild-to-moderate illness who were hospitalized in the Third People’s Hospital of Yangzhou from 7 to 20 August 2021 and analyzed the differences in symptoms and laboratory tests among fully vaccinated (FV), partially vaccinated (PV) and unvaccinated (UV) patients.

**Results:**

The median duration of viral RNA shedding was shorter in the FV (12 [IQR, 9.5–14] days) and PV (13 [IQR, 9–16.75] days) groups than in the UV group (15 [IQR, 11.75–17.25] days) (adjusted *P* < 0.001 and adjusted *P* = 0.23, respectively). The median titers of SARS-CoV-2-specific IgG and IgM were significantly higher in the FV (12.29 S/co [IQR, 2.08–63.59] and 0.3 S/co [IQR, 0.05–2.29], respectively) and PV (0.68 S/co [IQR, 0.14–28.69] and 0.12 S/co [0.03–5.23], respectively) groups than in the UV group (0.06 S/co [IQR, 0.03–0.47] and 0.04 S/co [IQR, 0.02–0.07]) (adjusted *P* < 0.001 and adjusted *P* = 0.008, respectively).

**Conclusions:**

Inactivated vaccines may shorten viral RNA shedding in breakthrough infected patients who have mild-to-moderate illness and may improve the ability of the host to generate specific antibodies to infection.

**Supplementary Information:**

The online version contains supplementary material available at 10.1186/s12879-022-07808-2.

## Introduction

Severe acute respiratory syndrome coronavirus 2 (SARS-CoV-2) variant infections are rising rapidly worldwide [1, 2]. The two SARS-CoV-2 mRNA vaccines, BNT162b2 and mRNA-1273, have been 94%-95% effective in preventing symptomatic coronavirus disease 2019 (COVID-19). However, there was a certain degree of reduction in the effectiveness of the vaccines in populations with comorbidities [3–6]. CoronaVac is an inactivated SARS-CoV-2 vaccine with a variable effectiveness rate, ranging from 50.65% to 83.50%, against symptomatic COVID-19 [7, 8]. Mutations in the spike (S) gene of SARS-CoV-2 variants, especially at neutralizing antibody-binding sites, result in the most severe reduction in the neutralization ability induced by mRNA and inactivated vaccines [9–11]. A test negative case‒control design to estimate the effectiveness of the BNT162b2 and ChAdOx1nCoV-19 vaccines against symptomatic disease caused by the delta variant found that the effectiveness of two doses were 88.0% and 67.0%, respectively [12]. A test negative case‒control study in Brazil showed that the effective rates of CoronaVac in older adults against symptomatic disease and death caused by the gamma mutant were 59% and 83.9%, respectively [13]. In Qatar, the effective rates of BNT162b2 and mRNA-1273 against infection with the delta variant were 51.9% and 73.1%, respectively [14]. Nevertheless, compared with unvaccinated (UV) individuals, the hospitalization rate and COVID-19-related mortality of fully vaccinated (FV) individuals were significantly lower [13, 15, 16].

At present, the effectiveness of inactivated vaccines for COVID-19 in fully immunized patients in a real-world population is poorly understood. Hence, there is an urgent need to elucidate the role of inactivated vaccines among breakthrough infections. The duration of viral RNA shedding is critical for determining therapeutic strategies to manage the disease and for making decisions regarding the patient’s discharge. To date, little information is available on the effect of inactivated vaccines on viral RNA shedding.

In this retrospective study, we reported the clinical characteristics of 147 hospitalized patients with COVID-19 to evaluate the role of inactivated vaccines on viral RNA shedding and to analyze laboratory tests of patients to optimize the clinical management of breakthrough infections.

## Methods

### Data collection

We conducted a retrospective study of 147 COVID-19 patients with mild to moderate illness who were hospitalized in the Third People’s Hospital of Yangzhou from 7 to 20 August 2021. Demographic and clinical variables were obtained from electronic records. Because our research was retrospective and noninterventional, it was granted an ethics exemption from the ethics committee of the Yangzhou Third People’s Hospital. SARS-CoV-2 infection was confirmed by a positive SARS-CoV-2 RNA test from nasopharyngeal swabs using a reverse transcription quantitative PCR method according to the manufacturer’s protocol (Liferiver Co. Ltd, Shanghai, China). Nasopharyngeal swab SARS-CoV-2 RNA test results, SARS-CoV-2 nucleocapsid gene cycle threshold (ct) values, complete blood counts, coagulation profiles, hypersensitive C-reactive protein (Hs-CRP) levels, serum biochemical parameters, interleukin-6 (IL-6) levels, chest computed tomography (CT), and levels of specific serum IgM and IgG antibodies of SARS-CoV-2 (Autobio Co. Ltd, Zhengzhou, China) were summarized on the day of admission. The nasopharyngeal swab samples were obtained by a medical team of 14 doctors (Chuancai Xu, Zhisong He, Wei Lei, Li Chen, Dan Shen, Xiaofei Wang, Deyu Xu, Jinzhou Zhu, Jindan Kong, Yao Wei, Daguo Zhao, Ting Xue, Ying Xu, Huayu Xu). According to Chinese COVID-19 guidelines (National Health Commission & State Administration of Traditional Chinese Medicine of China, 2020), the patients’ severities were classified into four categories: 1) mild: SARS-CoV-2 virus detection positive but without clinical symptoms and pneumonia or mild symptoms without pneumonia; 2) moderate: respiratory tract symptoms with pneumonia; 3) severe (fulfilled any of the following three criteria: respiratory distress, respiratory rate = 30 times/min; oxygen saturation = 93% in a resting state; arterial blood oxygen partial pressure (PaO_2_)/oxygen concentration (FiO_2_) = 300 mmHg; progressive worsening of clinical symptoms and the lung lesions having progressed by more than 50% within 24–48 h); and 4) critical (fulfilled any of the following three criteria: respiratory failure requiring mechanical ventilation, development of shock, and admission to ICU with other organ failure). Due to the fewer severe and critically ill COVID-19 patients treated in Yangzhou and the incomplete clinical data of these patients, only mild and moderate patients were included in this study. Breakthrough infection was defined as the detection of SARS-CoV-2 on an RT-PCR assay performed 14 or more days after receiving a second dose of an inactivated vaccine [17, 18].

In this study, viral RNA tests were repeated every 3 days on nasopharyngeal swab samples, starting on the day of admission. The patients who had been ill for more than 10 days, had a Ct value > 30, or had a negative viral RNA test one time were checked for viral RNA by nasopharyngeal swab testing every day.

### Inactivated vaccines vaccination

CoronaVac (600 SU per 0.5 ml of aluminum hydroxide per injection, Sinovac Life Sciences, Beijing, China) or BBIBP-CorV (6.5 U per 0.5 ml of aluminum hydroxide per injection, Beijing Institute of Biology, Beijing) was administered intramuscularly to those who were willing to be vaccinated. A total of 101 of the 147 patients had been vaccinated. Among them, 73 patients were FV who received two doses of inactivated vaccine with a two-to-four-week interval between doses, and 28 patients were partially vaccinated (PV) who received one dose inactivated vaccine (see Additional file 1: Table S1). The illness onset of all the vaccinated patients was more than 14 days after they had received the last dose of the vaccine.

### Chest CT scans

All patients underwent CT scanning (HiSpeed Advantage; GE Medical Systems, Milwaukee, Wis) with the following parameters: a 1.0 mm section thickness with a 6 mm gap, 1 s scanning time per section, 120 kV, and 214 mA. The images were taken using lung (window width, 1500 HU; window level, -700 HU) and mediastinal (window width, 350 HU; window level, 40 HU) settings. According to Chinese COVID-19 guidelines (National Health Commission & State Administration of Traditional Chinese Medicine of China, 2020), SARS-CoV-2 pneumonia was diagnosed in patients with patchy shadow or ground-glass opacities with or without consolidation on chest CT. All patients underwent chest CT examinations every three days after admission to check for the presence of SARS-CoV-2 pneumonia.

### Shedding duration

The day of viral RNA clearance was defined as the date of the first negative test when there were two sequential negative results for SARS-CoV-2 RNA from nasopharyngeal swab samples with more than a 24-h interval between them. The shedding duration was defined as the interval from illness onset to the date of the first negative SARS-CoV-2 RNA result from nasopharyngeal swab samples. Illness onset was defined as the first onset of symptoms or the first RT‒PCR positive result for the virus in asymptomatic patients.

### Anti-SARS-CoV-2 Spike IgG and IgM antibody titers assays

Three milliliters of blood were collected from the participants after they fasted for 6 h and was placed in coagulation tubes. The blood samples were centrifuged at 3,000 × g, and the upper serum layer was analyzed for anti-SARS-CoV-2 spike IgG and IgM antibodies using a chemiluminescent particle immunoassay with the Autolumo A2000 plus system (Autobio Co. Ltd, Zhengzhou, China) according to the manufacturer’s instructions.

### Treatment

The patients with lymphocyte count less than 0.8 × 10^9^/L were given 1.6 mg of thymosin α1 (Chengdu DIAO Pharmaceutical company, Chengdu, China) as an injection until their lymphocyte count increased to within the normal range. Each patient was treated with the traditional Chinese medicine Qingfei Paidu Decoction orally.

### Statistical analysis

New serological biomarkers, including neutrophil–lymphocyte ratio (NLR), platelet-lymphocyte ratio (PLR), lymphocyte-monocyte ratio (LMR), derived neutrophil–lymphocyte ratio (dNLR), high sensitivity C-reactive protein-albumin ratio (HsCAR), albumin-to-fibrinogen ratio (AFR), prognostic nutritional index (PNI), systemic immune-inflammation index (SII), and high sensitivity C-reactive protein-prealbumin ratio (HsCPAR), were calculated using formulas (see Additional file 2: Table S2). Continuous variables with a normal distribution are presented as the mean ± standard deviation and were analyzed by variance analysis, whereas continuous variables with a nonnormal distribution are expressed as the median with interquartile range (IQR) and were analyzed with Kruskal‒Wallis test. Categorical variables were expressed as the number and percentage and compared using the chi-square (χ^2^) test or Fisher’s exact test. Correlation analysis was performed using the Spearman method. The Spearman correlation coefficient was used to assess the associations of the variables with different indices. Kaplan‒Meier survival analysis was used to estimate the cumulative SARS-CoV-2 RNA-negativity rate. The statistical analysis was performed using IBM SPSS Statistics version 26.0 (IBM Corp., Armonk, NY, USA) or GraphPad Prism 8.0(GraphPad, San Diego, CA, USA). A *P* value of less than 0.05 (two-tailed) was considered statistically significant.

## Results

### Patient characteristics

The demographic, clinical and laboratory characteristics of the patients are summarized in Table 1 and Additional file 3: Table S3. Among the 147 patients, 65 patients were males (44.2%), and 82 patients were females (55.8%). The median age of the patients was 53 (IQR, 35–61) years. Forty-one patients (27.9%) were aged ≥ 60 years. Cough was most common symptom and was present in 42.9% of the patients on admission, followed by fever (30.6%). Asymptomatic patients accounted for 17.7% of the patients. Sore throat (15.6%), fatigue (8.8%), stuffy nose (8.2%) and runny nose (7.5%) were also frequently observed. The median time from illness onset to hospital admission was 2 days (IQR, 1–3 days). Fifty-four (36.7%) patients had comorbidities. The common comorbidities included hypertension (40 patients, 27.2%), diabetes (10 cases, 6.8%), and coronary heart disease (9 cases, 6.1%). There were 32 patients (21.8%) with liver or renal dysfunction. The median duration of viral RNA shedding was 13 days (IQR, 10–16). The average Ct value was 29.48 ± 5.99.

**Table 1:** Clinical Characteristics of 147 Hospitalized Patients

	Total (n = 147)	UV (n = 46)	PV (n = 28)	FV (n = 73)	*P*
Gender					0.524
Male, No. (%)	65 (44.2)	20 (43.5)	15 (53.6)	30 (41.1)	
Female, No. (%)	82 (55.8)	26 (56.5)	13 (46.4)	43 (58.9)	
Age, years	53 (35–61)	55.5 (32.5–69.25)	57 (38.5–65)	47 (35.5–58)	0.069
BMI, kg/m^2^	22.2 (20.83–24.74)	21.88 (20.74–24.77)	22.69 (20.61–24.72)	22.2 (20.9–24.56)	0.885
Current Smoking, No. (%)	61 (41.5)	18 (39.1)	14 (50)	29 (39.7)	0.596
Symptoms					
Fever, No. (%)	45 (30.6)	12 (26.1)	9 (32.1)	24 (32.9)	0.722
Cough, No. (%)	63 (42.9)	19 (41.3)	11 (39.3)	33 (45.2)	0.837
Diarrhea, No. (%)	6 (4.1)	2 (4.3)	1 (3.6)	3 (4.1)	
Sore throat, No. (%)	23 (15.6)	5 (10.9)	5 (17.9)	13 (17.8)	0.561
Chills, No. (%)	1 (0.7)	0	1 (3.6)	0	
Abdominal pain, No. (%)	1 (0.7)	1 (2.2)	0	0	
Loss of smell, No. (%)	3 (2)	0	1 (3.6)	2 (2.7)	
Loss of taste, No. (%)	2 (1.4)	0	0	2 (2.7)	
Muscle pains, No. (%)	4 (2.7)	3 (6.5)	1 (3.6)	0	
Loss of appetite, No. (%)	2 (1.4)	1 (2.2)	0	1 (1.4)	
Dizziness, No. (%)	1 (0.7)	1 (2.2)	0	0	
Fatigue, No. (%)	13 (8.8)	4 (8.7)	6 (21.4)	3 (4.1)	0.023
Stuffy nose, No. (%)	12 (8.2)	4 (8.7)	1 (3.6)	7 (9.6)	0.606
Runny nose, No. (%)	11 (7.5)	3 (6.5)	2 (7.1)	6 (8.2)	0.940
Chest tightness, No. (%)	1 (0.7)	1 (2.2)	0	0	
Headache, No. (%)	3 (2)	2 (4.3)	1 (3.6)	0	
Asymptomatic, No. (%)	26 (17.7)	10 (21.7)	5 (17.9)	11 (15.1)	0.649
Comorbidities, No. (%)	54 (36.7)	22 (47.8)	13 (46.4)	19 (26)	0.028
Hypertension, No. (%)	40 (27.2)	16 (34.8)	10 (35.7)	14 (19.2)	0.094
Diabetes, No. (%)	10 (6.8)	4 (8.7)	1 (3.6)	5 (6.9)	
Coronary heart disease, No. ()	9 (6.1)	4 (8.7)	1 (3.6)	4 (5.5)	
Chronic pulmonary disease, No. (%)	3 (2)	1 (2.2)	1 (3.6)	1 (1.4)	
Chronic liver disease, No. (%)	1 (0.7)	0	0	1 (1.4)	
Cancer, No. (%)	2 (1.4)	1 (2.2)	1 (3.6)	0	
Chronic kidney disease, No. (%)	2 (1.4)	1 (2.2)	1 (3.6)	0	
Hypothyroidism, No. (%)	1 (0.7)	1 (2.2)	0	0	
Rheumatoid Arthritis, No. (%)	1 (0.7)	1 (2.2)	0	0	
History of cerebral hemorrhage, No. (%)	2 (1.4)	1 (2.2)	0	1 (1.4)	
Arrhythmia, No. (%)	2 (1.4)	1 (2.2)	1 (3.6)	0	
Complications	32 (21.8)	15 (32.6)	5 (17.9)	12 (16.4)	0.098
Liver dysfunction, No. (%)	49 (33.3)	19 (41.3)	6 (21.4)	24 (32.9)	0.211
Renal dysfunction, No. (%)	55 (37.4)	21 (45.7)	11 (39.3)	23 (31.5)	0.292
Treatment					
Thymosin α1, No. (%)	32 (21.8)	7 (15.2)	5 (17.9)	20 (27.4)	0.250
Days from illness onset to hospital admission	2 (1–3)	2 (1–3)	2.5 (1–4)	2 (1–3)	0.336
Ct value	29.48 ± 5.99	27.84 ± 5.44	29.48 ± 6.55	30.52 ± 5.94	0.057

Data are presented as median (interquartile range) or mean ± standard deviation or the number (percentage). Continuous variables were analyzed by variance analysis or Kruskal‒Wallis test. Categorical variables were compared using the chi-square (χ^2^) test or Fisher’s exact test. A *P* value of less than 0.05 (two-tailed) was considered statistically significant

*FV* fully vaccinated, *PV* partially vaccinated, *UV* unvaccinated, *ct* SARS-CoV-2 nucleocapsid gene cycle threshold (ct) value

### Laboratory abnormalities

The median values of IL-6 and Hs-CRP were 12.70 (IQR, 7.20–23.2) pg/ml and 10.9 (IQR, 2–26.4) mg/L, respectively, which was higher than the upper limit of the normal range. The median lymphocyte count was 1.01 (IQR, 0.8–1.47) × 10^9^/L, which was lower than the lower limit of the normal range. The median values of D-dimer and lactate dehydrogenase (LDH) were 0.33 (IQR, 0.2–0.5) mg/L and 196 (IQR, 163–232) IU/L, respectively, and both were within the normal range (see Additional file 3: Table S3).

### Clinical and laboratory characteristics of the three groups

There were no significant differences in age, BMI, the percentage of comorbidities, or the median time from illness onset to hospital admission among the FV, PV and UV groups (see Table 1). There were no significant differences in the levels of IL-6, lymphocyte count, or LDH among the three groups. There were no statistically significant differences in the serological biomarkers, including LMR, NLR, PLR, dNLR, SII, AFR, HsCAR, HsCPAR and PNI, among the three groups (see Additional file 3: Table S3). The differences in the proportion of patients with abnormal levels of IL-6, lymphocyte count, LDH, and D-dimer in the three groups did not reach statistical significance (see Additional file 4: Table S4).

### The Duration of viral RNA shedding, Ct value, and the titers of specific IgG and IgM in the three groups

**Fig. 1:**
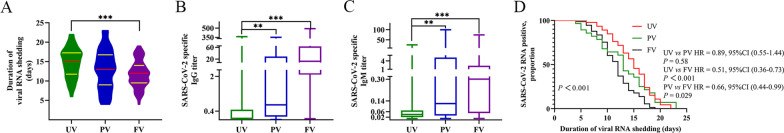
The duration of viral RNA shedding and the titers of specific IgG and IgM in the three groups. **A** The median duration of viral RNA shedding in the FV group was significantly lower than in the UV group (adjusted *P* < 0.001). The median duration of viral RNA shedding in the PV group lower than in the UV group, although the difference was not statistically significant (adjusted *P* = 0.23). **B** The median titers of SARS-CoV-2-specific IgG were significantly higher in the FV and PV groups than in the UV group (adjusted *P* < 0.001 and adjusted *P* = 0.008, respectively). **C** The median titers of SARS-CoV-2 specific-IgM were significantly higher in the FV and PV groups than in the UV group (adjusted *P* < 0.001 and adjusted *P* = 0.008, respectively). **D** Cumulative proportion of patients with detectable SARS-CoV-2 RNA by days after the illness onset between the UV, PV, and FV groups. Continuous variables were analyzed by variance analysis or Kruskal‒Wallis test. Kaplan‒Meier survival analysis was used to estimate the cumulative SARS-CoV-2 RNA negativity rate. A *P* value of less than 0.05 (two-tailed) was considered statistically significant. *FV* fully vaccinated, *PV* partially vaccinated, *UV* unvaccinated, *SARS-CoV-2* Severe Acute Respiratory Syndrome Coronavirus 2, *IQR* interquartile range

Our research found that the median duration of viral RNA shedding in the FV group was 12 (IQR, 9.5–14) days, which was significantly lower than the 15 (IQR, 11.75–17.25) days in the UV group (adjusted *P* < 0.001). The median duration of viral RNA shedding in the PV group was 13 (IQR, 9–16.75) days, which was lower than that in the UV group; however, the difference was not statistically significant (adjusted *P* = 0.23) (see Fig. 1A). The median titers of SARS-CoV-2 specific IgG were significantly higher in the FV (12.29S/co [IQR, 2.08–63.59]) and PV (0.68S/co [IQR, 0.14–28.69]) groups than in the UV group (0.06S/co [IQR, 0.03–0.47]) (adjusted *P* < 0.001 and adjusted *P* = 0.008, respectively) (see Fig. 1B). The median titers of SARS-CoV-2 specific IgM were significantly higher in the FV (0.3S/co [IQR, 0.05–2.29]) and PV (0.12S/co [0.03–5.23]) groups than in the UV group (0.04S/co [IQR, 0.02–0.07]) (adjusted *P* < 0.001 and adjusted *P* = 0.008, respectively) (see Fig. 1C). Kaplan‒Meier curve analysis showed that the FV group achieved a higher probability of faster viral RNA clearance than the UV group (*P* < 0.001, HR = 0.51, 95% CI [0.36–0.73]) and the PV group (*P* = 0.029, HR = 0.66, 95%CI [0.44–0.99]) (see Fig. 1D).

The average Ct value was higher in the FV (30.52 ± 5.94) and PV (29.48 ± 6.55) groups than in the UV group (27.84 ± 5.44), but the difference was not statistically significant (*P* = 0.057). Spearman correlation analysis found that the duration of viral RNA shedding was significantly negatively correlated with the Ct value (r = -0.249, *P* = 0.002) and the titers of SARS-CoV-2-specific IgG (r = -0.421, *P* < 0.001) and IgM (r = -0.341, *P* < 0.001). The Ct value was also significantly positively correlated with the titers of SARS-CoV-2-specific IgG (r = 0.338, *P* < 0.001) and IgM (r = 0.379, *P* < 0.001) (see Table 2 and Additional file 5: Table S5).

**Table2:** Viral RNA Shedding Duration Correlated with Ct Value, Specific IgG and IgM Titers

	Viral shedding duration after illness onset (days)	*P*
Ct value	r = − 0.249	0.002
SARS-CoV-2 specific IgG titer	r = − 0.421	0
SARS-CoV-2 specific IgM titer	r = − 0.341	0

Correlation analysis was performed using the Spearman method. Spearman correlation coefficient was represented by the r value. A *P* value of less than 0.05 (two-tailed) was considered statistically significant

ct, SARS-CoV-2 nucleocapsid gene cycle threshold (ct) value

### The duration of viral RNA shedding, Ct value, and the titers of specific IgG and IgM in the three groups with comorbidities

**Fig. 2:**
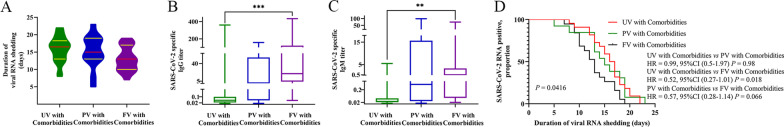
The duration of viral RNA shedding and the titers of specific IgG and IgM in the three groups with comorbidities. **A** The average duration of viral RNA shedding was lower in the FV and PV groups with comorbidities than in the UV group with comorbidities with no statistical significance (*P* = 0.07). **B** The median titer of SARS-CoV-2-specific IgG was significantly higher in the FV group with comorbidities than in the UV group with comorbidities (adjusted *P* < 0.001). The median titer of SARS-CoV-2-specific IgG was higher in the PV group with comorbidities than in the UV group with comorbidities with no statistical significance (adjusted *P* = 0.064). **C** The median titer of SARS-CoV-2-specific IgM was significantly higher in the FV group with comorbidities than in the UV group with comorbidities (adjusted *P* = 0.005). The median titer of SARS-CoV-2-specific IgM was higher in the PV group with comorbidities than in the UV group with comorbidities, although the difference was not statistically significant (Adjusted *P* = 0.056). **D** Cumulative proportion of patients with detectable SARS-CoV-2 RNA by days after the illness onset between the UV, PV, and FV groups with comorbidities. Continuous variables were analyzed by variance analysis or Kruskal‒Wallis test. Kaplan‒Meier survival analysis was used to estimate the cumulative SARS-CoV-2 RNA negativity rate. A *P* value of less than 0.05 (two-tailed) was considered statistically significant. *FV* fully vaccinated, *PV* partially vaccinated, *UV* unvaccinated, *SARS-CoV-2* Severe Acute Respiratory Syndrome Coronavirus 2, *IQR* interquartile range

We analyzed the clinical characteristics of 54 patients with comorbidities (see Table 3 and Additional file 6: Table S6) and found that the average duration of viral RNA shedding was still shorter in the FV and PV groups with comorbidities (13 ± 3.53 days and 15 ± 4.86 days, respectively) than in the UV group with comorbidities (15.91 ± 3.74 days), and there was no statistical significance (*P* = 0.07) (see Fig. 2A). The average Ct values of the FV, PV and UV groups with comorbidities were 28.68 ± 4.68, 30.37 ± 7.26, and 28.77 ± 5.5, respectively. There was no significant difference among the three groups with comorbidities (*P* = 0.665). The median titer of SARS-CoV-2-specific IgG was significantly higher in the FV group with comorbidities (19.73S/co [IQR, 3.74–120.4]) than in the UV group with comorbidities (0.05S/co [IQR, 0.03–0.1]) (adjusted *P* < 0.001). The median titer of SARS-CoV-2-specific IgG was higher in the PV group with comorbidities (1.4S/co [IQR, 0.05–51.86]) than in the UV group with comorbidities, and there was no statistical significance (adjusted *P* = 0.064) (see Fig. 2B). The median titer of SARS-CoV-2-specific IgM was significantly higher in the FV group with comorbidities (0.55S/co [IQR, 0.08–3.35]) than in the UV group with comorbidities (0.03S/co [IQR, 0.03–0.08]) (adjusted *P* = 0.005). The median titer of SARS-CoV-2-specific IgM was higher in the PV group with comorbidities (0.27S/co [IQR, 0.04–15.66]) than in the UV group with comorbidities, but the difference was not statistically significant (adjusted *P* = 0.056) (see Fig. 2C). Kaplan‒Meier curve analysis showed that the FV group with comorbidities achieved a higher probability of faster viral RNA clearance than the UV group with comorbidities (*P* = 0.018, HR = 0.52, 95% CI [0.27–1.01]) (see Fig. 2D).

**Table 3:** Clinical Characteristics of the Three Groups with Comorbidities

	Total (n = 54)	UV (n = 22)	PV (n = 13)	FV (n = 19)	*P*
Gender					0.573
Male, No. (%)	24 (44.4)	8 (36.4)	7 (53.8)	9 (47.4)	
Female, No. (%)	30 (55.6)	14 (63.6)	6 (46.2)	10 (52.6)	
Age, years	61.11 ± 12.41	63.05 ± 15.43	64.92 ± 10.75	56.26 ± 7.67	0.096
BMI, kg/m^2^	23.21 (21.96–25.56)	23.34 (20.88–25.86)	22.72 (22.1–25.27)	23.53 (22.23–25.35)	0.705
Current Smoking, No. (%)	24 (60)	9 (56.3)	7 (70)	8 (57.1)	0.734
Symptoms					
Fever, No. (%)	14 (35)	3 (18.8)	3 (30)	8 (57.1)	0.112
Cough, No. (%)	21 (52.5)	11 (68.8)	3 (30)	7 (50)	0.28
Diarrhea, No. (%)	3 (7.5)	1 (6.3)	1 (10)	1 (7.1)	
Sore throat, No. (%)	5 (12.5)	1 (6.3)	1 (10)	3 (21.4)	
Abdominal pain, No. (%)	1 (2.5)	1 (6.3)	0	0	
Loss of smell, No. (%)	2 (5)	0	1 (10)	1 (7.1)	
Muscle pains, No. (%)	2 (5)	2 (12.5)	0	0 (0)	
Dizziness, No. (%)	1 (2.5)	1 (6.3)	0	0 (0)	
Fatigue, No. (%)	7 (17.5)	4 (25)	2 (20)	1 (7.1)	
Stuffy nose, No. (%)	4 (10)	3 (18.8)	0	1 (7.1)	
Runny nose, No. (%)	2 (5)	1 (6.3)	1 (10)	0	
Headache, No. (%)	2 (5)	1 (6.3)	1 (10)	0	
Asymptomatic, No. (%)	6 (15)	4 (25)	1 (10)	1 (7.1)	
Complications					
Liver dysfunction, No. (%)	25 (46.3)	12 (54.5)	5 (38.5)	8 (42.1)	0.589
Renal dysfunction, No. (%)	26 (48.1)	9 (40.9)	6 (46.2)	11 (57.9)	0.547
Treatment					
Thymosin α1, No. (%)	9 (16.7)	4 (18.2)	1 (7.7)	4 (21.1)	0.591
Days from illness onset to hospital admission	2 (1–4)	2 (1–3)	4 (1.5–6)	2 (2–3)	0.060
Ct value	29.12 ± 5.65	28.77 ± 5.5	30.37 ± 7.26	28.68 ± 4.68	0.665

Data are presented as median (interquartile range) or mean ± standard deviation or the number (percentage). Continuous variables were analyzed by variance analysis or Kruskal‒Wallis test. Categorical variables were compared using the chi-square (χ^2^) test or Fisher’s exact test. A *P* value of less than 0.05 (two-tailed) was considered statistically significant

*FV* fully vaccinated, *PV* partially vaccinated, *UV* unvaccinated, *ct* SARS-CoV-2 nucleocapsid gene cycle threshold (ct) value

### The duration of viral RNA shedding, Ct value, and the titers of specific IgG and IgM in the three groups with hypertension

**Fig. 3:**
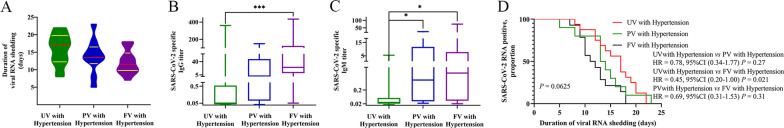
The duration of viral RNA shedding and the titers of specific IgG and IgM in the three groups with hypertension. **A.** The average duration of viral RNA shedding was lower in the FV and PV groups with hypertension than in the UV group with hypertension although the difference was not statistically significant (*P* = 0.065). **B.** The median titer of SARS-CoV-2-specific IgG was significantly higher in the FV group with hypertension than in the UV group with hypertension (adjusted *P* < 0.001). The median titer of SARS-CoV-2-specific IgG was higher in the PV group with hypertension than in the UV group with hypertension although the difference was not statistically significant (adjusted *P* = 0.113). **C.** The titers of SARS-CoV-2-specific IgM were significantly higher in the FV and PV groups with hypertension than in the UV group with hypertension (adjusted *P* = 0.035 and adjusted *P* = 0.027, respectively). **D.** Cumulative proportion of patients with detectable SARS-CoV-2 RNA by days after the illness onset between the UV, PV, and FV groups with hypertension. Continuous variables were analyzed by variance analysis or Kruskal‒Wallis test. Kaplan‒Meier survival analysis was used to estimate the cumulative SARS-CoV-2 RNA negativity rate. A *P* value of less than 0.05 (two-tailed) was considered statistically significant. *FV* fully vaccinated, *PV* partially vaccinated, *UV* unvaccinated; *SARS-CoV-2* Severe Acute Respiratory Syndrome Coronavirus 2, *IQR* interquartile range

We also analyzed patients with hypertension (see Table 4 and Additional file 7: Table S7) and found that the average duration of viral RNA shedding was lower in the FV and PV groups with hypertension (12.21 ± 3.49 days and 13.8 ± 4.96 days, respectively) than in the UV group with hypertension (15.94 ± 4.31 days), but the difference was not statistically significant (*P* = 0.065) (see Fig. 3A). The average Ct value was higher in the FV (29.87 ± 5.53) and PV (31.84 ± 7.22) groups with hypertension than in the UV group with hypertension (29.18 ± 6), but the difference was not statistically significant (*P* = 0.561). The median titer of SARS-CoV-2-specific IgG was significantly higher in the FV group with hypertension (24.81S/co [IQR, 9.85–123.09]) than in the UV group with hypertension (0.05S/co [IQR, 0.02–0.52]) (adjusted *P* < 0.001). The median titer of SARS-CoV-2-specific IgG was higher in the PV group with hypertension (2.09S/co [IQR, 0.11–46.77]) than in the UV group with hypertension, but the difference did not reach statistical significance (adjusted *P* = 0.113) (see Fig. 3B). The titers of SARS-CoV-2-specific IgM were significantly higher in the FV and PV groups with hypertension (0.42S/co [IQR, 0.06–7.83] and 0.33S/co [IQR, 0.05–11.75], respectively) than in the UV group with hypertension (0.04S/co [IQR, 0.02–0.1]) (adjusted *P* = 0.035 and adjusted *P* = 0.027, respectively) (see Fig. 3C). Kaplan‒Meier curve analysis showed that the FV group with hypertension achieved a higher probability of faster viral RNA clearance than the UV group with hypertension (*P* = 0.021, HR = 0.45, 95% CI [0.20–1.00]) (see Fig. 3D).

**Table 4:** Clinical Characteristics of the Three Groups with Hypertension

	Total (n = 40)	UV (n = 16)	PV (n = 10)	FV (n = 14)	*P*
Gender					1
Male, No. (%)	17 (42.5)	7 (43.8)	4 (40)	6 (42.9)	
Female, No. (%)	23 (57.5)	9 (56.3)	6 (60)	8 (57.1)	
Age, years	59 (54.5–66.5)	62 (53.25–75.75)	60 (56.75–69.5)	59 (52.75–61)	0.4
BMI, kg/m^2^	24.08 ± 2.79	24.28 ± 2.93	24.24 ± 3.1	23.74 ± 2.57	0.858
Current Smoking, No. (%)	17 (42.5)	6 (37.5)	4 (40)	7 (50)	0.774
Symptoms					
Fever, No. (%)	9 (22.5)	2 (12.5)	1 (10)	6 (42.9)	0.077
Cough, No. (%)	16 (40)	4 (25)	7 (70)	5 (35.7)	0.069
Diarrhea, No. (%)	4 (10)	2 (12.5)	0	2 (14.3)	
Sore throat, No. (%)	4 (10)	1 (6.3)	1 (10)	2 (14.3)	
Abdominal pain, No. (%)	1 (2.5)	1 (6.3)	0	0	
Muscle pains, No. (%)	1 (2.5)	1 (6.3)	0	0	
Dizziness, No. (%)	1 (2.5)	1 (6.3)	0	0	
Fatigue, No. (%)	5 (12.5)	3 (18.8)	2 (20)	0	
Stuffy nose, No. (%)	4 (10)	3 (18.8)	0	1 (7.1)	
Runny nose, No. (%)	1 (2.5)	1 (6.3)	0	0	
Headache, No. (%)	2 (5)	1 (6.3)	1 (10)	0 (0)	
Asymptomatic, No. (%)	5 (12.5)	3 (18.8)	1 (10)	1 (7.1)	
Complications					
Liver dysfunction, No. (%)	20 (50)	10 (62.5)	4 (40)	6 (42.9)	0.43
Renal dysfunction, No. (%)	17 (42.5)	7 (43.8)	3 (30)	7 (50)	0.615
Treatment					
Thymosin α1, No. (%)	8 (20)	3 (18.8)	1 (10)	4 (28.6)	0.618
Days from illness onset to hospital admission	2 (1.25–4)	2 (1–3)	4 (1–6)	2 (2–3)	0.132
Ct value	30.09 ± 6.1	29.18 ± 6	31.84 ± 7.22	29.87 ± 5.53	0.561

Data are presented as median (interquartile range) or mean ± standard deviation or the number (percentage). Continuous variables were analyzed by variance analysis or Kruskal‒Wallis test. Categorical variables were compared using the chi-square (χ^2^) test or Fisher’s exact test. A *P* value of less than 0.05 (two-tailed) was considered statistically significant

*FV* fully vaccinated, *PV* partially vaccinated, *UV* unvaccinated, *ct* SARS-CoV-2 nucleocapsid gene cycle threshold (ct) value

### The duration of viral RNA shedding, intervals from virus shedding to the onset of pneumonia lesion reduction on CT, and the titers of specific IgG and IgM in the three groups with SARS-CoV-2 pneumonia

**Fig. 4:**
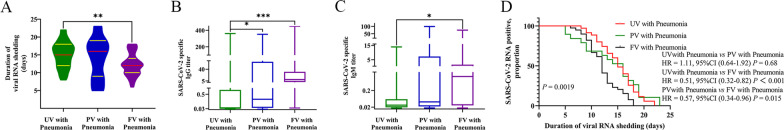
The duration of viral RNA shedding and the titers of specific IgG and IgM in the three groups with SARS-CoV-2 pneumonia. **A** The average duration of viral RNA shedding in the FV group with SARS-CoV-2 pneumonia was significantly lower than in the UV group with SARS-CoV-2 pneumonia (adjusted *P* = 0.002). The average duration of viral RNA shedding in the PV group with SARS-CoV-2 pneumonia was lower than in the UV group with SARS-CoV-2 pneumonia, although the difference was not statistically significant (adjusted *P* = 0.835). **B** The median titers of SARS-CoV-2-specific IgG were significantly higher in the FV and PV groups with SARS-CoV-2 pneumonia than in the UV group with SARS-CoV-2 pneumonia (adjusted *P* < 0.001 and adjusted *P* = 0.035, respectively). **C** The median titers of SARS-CoV-2 specific IgM were significantly higher in the FV group with SARS-CoV-2 pneumonia than in the UV group with SARS-CoV-2 pneumonia (adjusted *P* = 0.007). The titers of SARS-CoV-2 specific IgM were higher in the PV group with SARS-CoV-2 pneumonia than in the UV group with SARS-CoV-2 pneumonia, although the difference was not statistically significant (adjusted *P* = 0.068). **D** Cumulative proportion of patients with detectable SARS-CoV-2 RNA by days after illness onset between the UV, PV, and FV groups with SARS-CoV-2 pneumonia. Continuous variables were analyzed by variance analysis or Kruskal‒Wallis test. Kaplan‒Meier survival analysis was used to estimate the cumulative SARS-CoV-2 RNA negativity rate. A *P* value of less than 0.05 (two-tailed) was considered statistically significant. *FV* fully vaccinated, *PV* partially vaccinated, *UV* unvaccinated, *SARS-CoV-2* Severe Acute Respiratory Syndrome Coronavirus 2, *IQR* interquartile range

The average duration of viral RNA shedding in the FV group with SARS-CoV-2 pneumonia was 12.29 ± 3.14 days, which was significantly lower than the 15.16 ± 3.77 days in the UV group with SARS-CoV-2 pneumonia (adjusted *P* = 0.002). The average duration of viral RNA shedding in the PV group with SARS-CoV-2 pneumonia was 14.08 ± 5.64 days, which was lower than that in the UV group with SARS-CoV-2 pneumonia; however, the difference was not statistically significant (adjusted *P* = 0.835) (see Fig. 4A). The median titers of SARS-CoV-2-specific IgG were significantly higher in the FV (9.25S/co [IQR, 1.89–32.24]) and PV (0.34S/co [IQR, 0.06–62.05]) groups with SARS-CoV-2 pneumonia than in the UV group with SARS-CoV-2 pneumonia (0.06S/co [IQR, 0.03–0.64]) (adjusted *P* < 0.001 and adjusted *P* = 0.035, respectively) (see Fig. 4B). The median titers of SARS-CoV-2-specific IgM were significantly higher in the FV group with SARS-CoV-2 pneumonia (0.35S/co [IQR, 0.04–1.62]) than in the UV group with SARS-CoV-2 pneumonia (0.04S/co [IQR, 0.02–0.11]) (adjusted *P* = 0.007) (see Fig. 4C). Among the three groups, there were no difference in the time intervals from viral shedding to the day when SARS-CoV-2 pneumonia lesions began to decrease on CT images. Interestingly, the time intervals from viral shedding to the onset of reduction of pneumonia lesions on CT were within -3 to 3 days in 64.5% of the three groups (see Table 5). Kaplan‒Meier curve analysis showed that the FV group with SARS-CoV-2 pneumonia achieved a higher probability of faster viral RNA clearance than the UV group with SARS-CoV-2 pneumonia (*P* < 0.001, HR = 0.51, 95% CI [0.32–0.82]) and the PV group with SARS-CoV-2 pneumonia (*P* = 0.015, HR = 0.57, 95% CI [0.34–0.96]) (see Fig. 4D).

**Table 5:** Clinical Characteristics of the Three Groups with SARS-CoV-2 Pneumonia

	Total (n = 93)	UV (n = 35)	PV (n = 19)	FV (n = 39)	*P*
Gender					0.452
Male, No. (%)	42 (45.2)	15 (42.9)	11 (57.9)	16 (41)	
Female, No. (%)	51 (54.8)	20 (57.1)	8 (42.1)	23 (59)	
Age, years	53 ± 16.37	55.63 ± 20.04	55.32 ± 17.53	49.51 ± 11.06	0.219
BMI, kg/m^2^	22.77 ± 2.93	23.22 ± 3.56	21.76 ± 2.58	22.86 ± 2.36	0.211
Smoking, No. (%)	37 (39.8)	12 (31.4)	8 (42.1)	18 (46.2)	0.422
Comorbidities, No. (%)	54 (58.1)	25 (71.4)	11 (57.9)	18 (46.2)	0.089
Treatment					
Thymosin α1, No. (%)	22 (23.7)	6 (16.7)	3 (15.8)	13 (33.3)	0.174
Days from illness onset to hospital admission	2 (1–3)	2 (1–3)	2 (1–4)	2 (1–3)	0.880
Ct value	28.98 ± 5.93	27.68 ± 5.45	29.15 ± 6.92	30.07 ± 5.75	0.225
IVOR, days	1 (− 1–4)	2 (− 1–5)	0 (-1–4)	1 (− 2–4)	0.555
IL-6, pg/ml	14.6 (8.3–28.2)	13.4 (8.2–28.8)	19.3 (13.2–34.4)	13.9 (8–25.3)	0.454
IVORD, No. (%)	60 (64.5)	21 (60)	14 (73.7)	25 (64.1)	0.603

Data are presented as median (interquartile range) or mean ± standard deviation or the number (percentage). Continuous variables were analyzed by variance analysis or Kruskal‒Wallis test. Categorical variables were compared using the chi-square (χ^2^) test or Fisher’s exact test. A *P* value of less than 0.05 (two-tailed) was considered statistically significant

*FV* fully vaccinated, *PV* partially vaccinated, *UV*,= unvaccinated, *ct* SARS-CoV-2 nucleocapsid gene cycle threshold (ct) value, *IVOR* the time interval from viral RNA shedding to the onset of pneumonia lesion reduction on CT, *IL-6* interleukin-6, *IVORD* the time interval from viral RNA shedding to the onset of pneumonia lesion reduction on CT within − 3 to 3 days

## Discussion

Our study was the first to demonstrate that inactivated vaccines may shorten viral RNA shedding in COVID-19 patients with mild-to-moderate illness.

This study found that the duration of viral RNA shedding was shorter in the FV and PV groups than in the UV group, especially the FV group. Previous studies have found that prolonged viral RNA shedding was related to old age, hypertension, male sex, delayed admission to hospital after illness onset, diarrhea, corticosteroid therapy, invasive mechanical ventilation, and low lymphocyte count [19, 20]. There were no significant differences in these factors among the three groups in our study.

Our study also found that the titers of SARS-CoV-2-specific IgG and IgM were significantly higher in the PV and FV groups than that in the UV group. We found that the duration of viral RNA shedding was negatively correlated with the titers of SARS-CoV-2-specific IgG and IgM. In our study, the SARS-CoV-2-specific IgG and IgM antibody titers were measured after infection, and the median time from illness onset to hospital admission of all patients was 2 days. The UV patients had lower median titers of both antibodies, suggesting that these patients may have a lower immune response capacity to infection in the early stage.

FV subjects have a strong SARS-CoV-2-specific CD4 + and CD8 + T-cells response [21] and a large number of SARS-CoV-2 spike protein-targeting B cells in their lymph node germinal center [22]. Akiko Iwasaki et al. found increased levels of activated CD4 + T cells, follicular helper T cells, and antibody-secreting cells post vaccination [9]. Another study found that vaccine-generated cells exhibited a consistent antiviral functional profile, including IFN-γ expression, spike-specific memory CD4 + and CD8 + T-cells, and the presence of multicytokine-expressing cells after a second low-dose mRNA-1273 immunization [23]. It was reported that IgG antibodies against the N protein and CD4 + T-cell responses stimulated by the MegaPool (MP) peptide of the “nonspike” SARS-CoV-2 proteome were detected after two doses of the CoronaVac vaccine [24, 25]. One study found that both the BNT162b2 and CoronaVac vaccines induced SARS-CoV-2-specific CD4 + and CD8 + T-cell responses, but CoronaVac elicited significantly higher structural protein-specific CD4 + and CD8 + T-cell responses [26]. These studies have shown that the neutralizing antibodies in FV patients gradually decline, but the immune memory persists. Therefore, the ability for viral clearance improves. This may explain the shorter viral RNA shedding time and higher specific IgG and IgM levels of the vaccinated patients in our study.

We found that the duration of viral RNA shedding was related to the Ct value. The Ct value represents the viral load. The higher the Ct value, the lower the viral load. The Ct value of the vaccinated patients was higher than that of the unvaccinated patients, which was also the reason for the shorter viral RNA clearance time of the vaccinated patients.

A retrospective multicenter cohort study in the United States analyzed 1305 inpatients with COVID-19 and found that 72.6% of the patients had at least one comorbidity, among which hypertension (56.2%) and diabetes (30.1%) were the most common, and comorbidity was an independent predictor of in-hospital mortality [27]. Another multicenter retrospective study found that patients with comorbidities were older and more likely to have abnormal chest radiographic manifestations, and the prognosis of these patients was worse [28]. Our research found that patients with comorbidities were older, and the most common comorbidities were hypertension and diabetes. These results were consistent with the studies mentioned above.

A study found that lower antibody concentrations were consistently associated with older age and comorbidities, including diabetes, hypertension, heart disease, and autoimmune diseases, after vaccination with the BNT162b2 vaccine [29]. Another study found that older individuals (aged ≥ 60 years) or people with diabetes or chronic diseases had a significantly lower SARS-CoV-2 IgG antibody response after the first and second doses of CoronaVac vaccine than young and healthy subjects did [30]. This difference may indicate that breakthrough infections are more likely to occur in older patients with comorbidities, including hypertension and diabetes [31]. We found that vaccinated patients with comorbidities had significantly higher titers of SARS-CoV-2-specific IgG and IgM after infection than UV patients with comorbidities. We also found that vaccinated patients with comorbidities had a shorter duration of viral RNA shedding than UV patients with comorbidities did. However, the difference was not statistically significant. Similar results were also seen in the groups with hypertension. These results indicated that inactivated vaccines may improve the early immune response in infected patients with comorbidities (including hypertension). However, the degree of improvement was not as obvious as that in the patients without comorbidities.

We also observed that the duration of viral RNA shedding was shorter and the SARS-CoV-2-specific IgG and IgM titers were higher in the FV group with SARS-CoV-2 pneumonia. Interestingly, the time intervals from viral RNA shedding to the onset of pneumonia lesion reduction on CT images ranged mostly from -3 to 3 days in all of the SARS-CoV-2 pneumonia patients. More in-depth studies are needed to elucidate the mechanism underlying this phenomenon.

This study has some limitations. First, all 147 patients in this study accounted for the majority of the patients who were treated at the Third People's Hospital of Yangzhou. Patients with incomplete data were excluded. Thus, these data are representative of patients with mild to moderate illness. Second, since there were fewer critically ill patients in Yangzhou and only a few critically ill patients were vaccinated, only mild-to-moderate patients were included in our study, which does not mean that an inactive vaccine could reduce the duration of viral RNA shedding in critically ill patients. Third, specifically, our findings concerning SARS-CoV-2-specific IgG and IgM antibody titers do not necessarily represent the titers achieved although vaccination and cannot be used to estimate the correlation of protection. Fourth, since nearly all the patients were given traditional Chinese medicine treatment, we were not able to judge whether these treatments had any effect on viral RNA shedding.

## Conclusions

In summary, our research found that the duration of viral RNA shedding was shorter and that SARS-CoV-2-specific IgG and IgM titers were higher in vaccinated patients. Similar results were observed in the population of patients with comorbidities. These findings showed that inactivated vaccines had a certain protective effect on patients with COVID-19.

## Supplementary Information

**Additional file 1: Table S1.** Proportions of patients vaccinated with different inactivated vaccines.

**Additional file 2: Table S2. **Serological markers and their formulas.

**Additional file 3: Table S3.** Laboratory tests of the three groups.

**Additional file 4: Table S4. **Proportions of abnormal level of IL-6, lymphocyte count, LDH, and D-dimmer in the three groups.

**Additional file 5: Table S5. **Ct value correlated with SARS-CoV-2 specific IgG and IgM Titers.

**Additional file 6: Table S6. **Laboratory tests of the three groups with comorbidities.

**Additional file 7: Table S7.** Laboratory tests of the three groups with hypertension.

## Data Availability

The datasets used and/or analyzed during the current study are available from the corresponding author on reasonable request.

## Abbreviations

RNA: Ribonucleic acid
SARS-CoV-2: Severe acute respiratory syndrome Coronavirus-2
COVID-19: 2019 Coronavirus disease
FV: Fully vaccinated
PV: Partially vaccinated
UV: Unvaccinated
IQR: Interquartile range
IgG: Immunoglobulin G
IgM: Immunoglobulin M
RT-PCR: Reverse transcription- Polymerase chain reaction
Hs-CRP: Hypersensitive C-reactive protein
IL-6: Interlukin-6
CT: Chest computed tomography
NLR: Neutrophil–lymphocyte ratio
PLR: Platelet-lymphocyte ratio
LMR: Lymphocyte-monocyte ratio
dNLR: Derived neutrophil–lymphocyte ratio
HsCAR: High sensitivity C-reactive protein-albumin ratio
AFR: Albumin-to-fibrinogen ratio
PNI: Prognostic nutritional index
SII: Systemic immune-inflammation index
HsCPAR: High sensitivity C-reactive protein-prealbumin ratio
LDH: Lactate dehydrogenase
