# Supplementary material for: Comparison of the duration of viral RNA shedding and anti-SARS-CoV-2 spike IgG and IgM antibody titers in COVID-19 patients who were vaccinated with inactivated vaccines or not: a retrospective study
Source: BMC Infect Dis. 2022 Nov 9;22:831. doi: 10.1186/s12879-022-07808-2 (PMC9645737; doi:10.1186/s12879-022-07808-2)
Supplement: Supplementary file 1 — Additional file 1: Table S1. Proportions of patients vaccinated with different inactivated vaccines. [file 12879_2022_7808_MOESM1_ESM.docx]

**Additional file 1:** **Table S1. Proportion of Patients Vaccinated with Different Inactivated Vaccines**

|  | **Total (n = 101)** | **PV (n = 28)** | **FV (n = 73)** | ***P*** |
| --- | --- | --- | --- | --- |
| CoronaVac, No. (%) | 40 (39.6) | 11 (39.3) | 29 (39.7) | 0.968 |
| BBIBP-CorV, No. (%) | 40 (39.6) | 4 (14.3) | 36 (49.3) | 0.001 |
| Unknown*, No. (%) | 21 (20.8) | 13 (46.4) | 8 (11) | <0.001 |

*Unknown: It was not clear which of the two vaccines, CoronaVac and BBIBP-CorV, was vaccinated.

Data are presented as the number (percentage). Categorical variables were compared using the chi-square (χ²) test. A *P* value of less than 0.05 (two-tailed) was considered statistically significant.

**Abbreviations:** FV, fully vaccinated; PV, partially vaccinated.
