# Supplementary material for: Comparison of the duration of viral RNA shedding and anti-SARS-CoV-2 spike IgG and IgM antibody titers in COVID-19 patients who were vaccinated with inactivated vaccines or not: a retrospective study
Source: BMC Infect Dis. 2022 Nov 9;22:831. doi: 10.1186/s12879-022-07808-2 (PMC9645737; doi:10.1186/s12879-022-07808-2)
Supplement: Supplementary file 2 — Additional file 2: Table S2. Serological markers and their formulas. [file 12879_2022_7808_MOESM2_ESM.docx]

**Additional file 2: Table S2. Serological Markers and Their Formulas**

| **Indexes** | **Formulas** |
| --- | --- |
| NLR | NEU counts (10^9^)/LYM counts (10^9^) |
| PLR | PLT counts (10^9^)/LYM counts (10^9^) |
| LMR | LYM counts (10^9^)/MON counts (10^9^) |
| dNLR | NEU counts (10^9^)/ [WBC counts (10^9^)-NEU counts (10^9^)] |
| HsCAR | HsCRP (mg/L)/ALB (g/L) |
| AFR | ALB (g/L)/Fbg (g/L) |
| PNI | 10*ALB (g/L) + 5 * LYM counts (10^9^) |
| SⅡ | PLT counts (10^9^) * NEU counts (10^9^)/LYM counts (10^9^) |
| HsCPAR | HsCRP (mg/L)/PA (g/L) |

**Abbreviations:** NLR, neutrophil-lymphocyte ratio; PLR, platelet-lymphocyte ratio; LMR, lymphocyte-monocyte ratio; dNLR, derived neutrophil-lymphocyte ratio; HsCAR, high sensitivity C-reactive protein-albumin ratio; AFR, albumin-to-fibrinogen ratio; PNI, prognostic nutritional index; SⅡ, systemic immune-inflammation index; HsCPAR, high sensitivity C-reactive protein-prealbumin ratio.
