# Supplementary material for: Comparison of the duration of viral RNA shedding and anti-SARS-CoV-2 spike IgG and IgM antibody titers in COVID-19 patients who were vaccinated with inactivated vaccines or not: a retrospective study
Source: BMC Infect Dis. 2022 Nov 9;22:831. doi: 10.1186/s12879-022-07808-2 (PMC9645737; doi:10.1186/s12879-022-07808-2)
Supplement: Supplementary file 3 — Additional file 3: Table S3. Laboratory tests of the three groups. [file 12879_2022_7808_MOESM3_ESM.docx]

**Additional file 3: Table S3. Laboratory Tests of the Three Groups**

|  | **Total (n = 147)** | **UV (n = 46)** | **PV (n = 28)** | **FV (n = 73)** | ***P*** |
| --- | --- | --- | --- | --- | --- |
|  |  |  |  |  |  |
| IL-6, pg/ml | 12.70 (7.20 - 23.2) | 12.9 (7.5 - 28.15) | 14.1 (7.48 - 23.85) | 11.7 (6.55 - 21.85) | 0.579 |
| RDW-CV | 12.3 (11.9 - 12.7) | 12.35 (11.9 - 13.13) | 12.2 (11.7 - 12.65) | 12.2 (11.9 - 12.5) | 0.173 |
| NEU, ×109/l | 3.4 (2.64 - 4.41) | 3.14 (2.41 - 4.15) | 3.3 (2.73 - 4.68) | 3.5 (2.7 - 4.45) | 0.237 |
| MON, ×109/l | 0.5 (0.4 - 0.69) | 0.5 (0.4 - 0.7) | 0.44 (0.31 - 0.58) | 0.5 (0.4 - 0.7) | 0.272 |
| MPV, fL | 11.1 (10.5 - 12) | 10.9 (10.45 - 12) | 11.4 (10.7 - 11.98) | 11.1 (10.4 - 12.05) | 0.346 |
| MCHC, g/L | 341 (335 - 348) | 339.5 (334.75 - 346.5) | 343 (335 - 349.75) | 342 (335 - 349) | 0.574 |
| MCH, pg | 30.2 (29.3 - 31.3) | 30.05 (29.1 - 30.93) | 30.65 (30.03 - 31.53) | 30.1 (29.3 - 31.35) | 0.088 |
| LYM, ×109/l | 1.01 (0.8 - 1.47) | 1.1 (0.74 - 1.48) | 0.88 (0.72 - 1.36) | 1.01 (0.87 - 1.5) | 0.384 |
| WBC, ×109/l | 5.22 (4.18 - 6.43) | 4.88 (3.98 - 5.96) | 5.3 (4.19 - 6.42) | 5.4 (4.24 - 6.6) | 0.487 |
| RBC, ×1012/l | 4.51 ± 0.50 | 4.46 ± 0.53 | 4.36 ± 0.48 | 4.59 ± 0.48 | 0.091 |
| RDW-SD | 40.39 ± 4.02 | 40.06 ± 4.77 | 39.86 ± 4.17 | 40.80 ± 3.41 | 0.460 |
| MCV, fL | 88.2 (86.2 - 90.8) | 87.9 (85.55 - 90.85) | 89.7 (87 - 91.5) | 88.3 (86.1 - 90.35) | 0.250 |
| PLT, ×109/l | 183 (142 - 226) | 165 (137.5 - 202) | 177 (118.75 - 212.75) | 191 (160 - 234) | 0.057 |
| PCT | 0.2 (0.18 - 0.23) | 0.2 (0.17 - 0.21) | 0.2 (0.13 - 0.22) | 0.2 (0.2 - 0.27) | 0.147 |
| HB, g/L | 137 (123 - 149) | 132 (122.75 - 150.25) | 131.5 (123.25 - 146.25) | 140 (125.5 - 150.5) | 0.220 |
| NLR | 3.2 (2.2 - 4.5) | 2.9 (2.08 - 3.73) | 3.85 (2.63 - 5.55) | 3.2 (2.2 - 4.6) | 0.160 |
| LMR | 2.1 (1.5 - 3.1) | 2.1 (1.58 - 3.13) | 2.05 (1.5 - 2.88) | 2.1 (1.45 - 3.1) | 0.968 |
| PLR | 178.7 (129.6 - 221.4) | 158.75 (113.78 - 200.75) | 178.5 (129.65 - 225.98) | 193.9 (141.6 - 226.2) | 0.542 |
| dNLR | 2 (1.5 - 2.8) | 1.9 (1.38 - 2.43) | 2.4 (1.83 - 3.18) | 2 (1.55 - 2.8) | 0.076 |
| SⅡ | 547.4 (395 - 871.3) | 484.6 (328.75 - 667.33) | 526.3 (413.3 - 1029.73) | 672.2 (466.6 - 870.25) | 0.213 |
| AFR | 14.4 (11.8 - 17.9) | 15.4 (12.55 - 19.78) | 15.05 (11.83 - 17.45) | 13.6 (10.9 - 16.95) | 0.021 |
| D-dimer, mg/L | 0.33 (0.2 - 0.5) | 0.35 (0.2 - 0.5) | 0.33 (0.26 - 0.55) | 0.33 (0.2 - 0.51) | 0.927 |
| PT, s | 11.7 (11.3 - 12.4) | 11.8 (11.2 - 12.4) | 11.5 (11.2 - 11.88) | 11.9 (11.4 - 12.45) | 0.142 |
| PTA | 89.95 ± 13.49 | 90.05 ± 15.62 | 93.75 ± 10.27 | 88.43 ± 13.01 | 0.208 |
| TT, s | 17.9 (17.3 - 18.5) | 18.2 (17.78 - 18.73) | 17.95 (17.3 - 18.6) | 17.7 (17.1 - 18.2) | 0.001 |
| INR | 1 (1 - 1.1) | 1.02 (0.97 - 1.1) | 1 (0.99 - 1.03) | 1.04 (1 - 1.1) | 0.187 |
| AT Ⅲ | 91.37 ± 11.64 | 90.64 ± 13.33 | 94.54 ± 8.74 | 90.61 ± 11.42 | 0.278 |
| FDP, ug/ml | 2 (1.6 - 2.4) | 2.2 (1.82 - 2.59) | 2.01 (1.81 - 2.21) | 1.8 (1.44 - 2.21) | 0.012 |
| FBG, g/L | 3.19 (2.64 - 3.99) | 2.92 (2.47 - 3.71) | 3.09 (2.62 - 3.88) | 3.47 (2.85 - 4.15) | 0.012 |
| APTT, s | 29.1 (26.4 - 31.5) | 30 (27.4 - 33.4) | 29.3 (26.33 - 31.23) | 28.8 (25.75 - 31.1) | 0.174 |
| HBDH, U/L | 150 (125 - 172) | 152.5 (127.5 - 168.25) | 152.5 (129.75 - 181.25) | 145 (123.5 - 172) | 0.527 |
| LDH, IU/L | 196 (163 - 232) | 197 (168.5 - 217.25) | 198 (172.75 - 241.5) | 192 (161 - 235.5) | 0.656 |
| LDL, mmol/L | 2.17 (1.7 - 2.7) | 2.09 (1.51 - 2.56) | 2.19 (1.7 - 2.68) | 2.2 (1.8 - 2.8) | 0.173 |
| UA, μmol/L | 4.1 (3.3 - 5.1) | 4.4 (3.5 - 5.3) | 4.8 (3.35 - 5.2) | 3.8 (3.2 - 4.8) | 0.053 |
| CHOL, mmol/L | 3.9 (3.2 - 4.36) | 3.93 (3.17 - 4.27) | 4.07 (3.63 - 4.76) | 3.9 (3.2 - 4.32) | 0.273 |
| TP, g/L | 73.6 (68.8 - 78) | 74.4 (68.63 - 78.55) | 74.7 (68.35 - 78.65) | 72.8 (69.35 - 77.5) | 0.800 |
| GLB, g/L | 26.73 ± 4.41 | 27.07 ± 4.48 | 26.55 ± 5.28 | 26.58 ± 4.03 | 0.817 |
| TG, mmol/L | 1.1 (0.82 - 1.79) | 1 (0.87 - 1.83) | 1.22 (1 - 1.95) | 1.13 (0.8 - 1.7) | 0.278 |
| ALB, g/L | 47.1 (44.3 - 49.4) | 47.7 (43.68 - 49.55) | 46.95 (44.18 - 49.3) | 47.1 (44.3 - 49.3) | 0.903 |
| A/G | 1.7 (1.6 - 2) | 1.7 (1.5 - 2) | 1.7 (1.6 - 2.1) | 1.7 (1.6 - 2) | 0.962 |
| ALP, U/L | 81 (68 - 97) | 84.5 (71.5 - 105) | 87.5 (70 - 102.25) | 76 (63.5 - 93.5) | 0.161 |
| CREA, μmol/L | 68 (60 - 81) | 69.5 (64 - 84.75) | 71 (61.75 - 84.75) | 66 (56.5 - 75) | 0.055 |
| CK, U/L | 92 (60 - 134) | 96 (65.75 - 164.75) | 86 (53.25 - 129.75) | 91 (58 - 126) | 0.327 |
| CKMB, U/L | 12.7 (9.9 - 16) | 13.35 (10.38 - 16.2) | 10.8 (8.53 - 13.55) | 13.1 (10.1 - 16.25) | 0.064 |
| LPA, mg/L | 78.8 (34.2 - 237.5) | 57.3 (31.48 - 143.75) | 102.3 (35.35 - 327.33) | 72.9 (35.45 - 248.6) | 0.280 |
| ADA, U/L | 13 (11 - 16) | 15 (12 - 17) | 13 (11 - 15.75) | 13 (10.5 - 15) | 0.071 |
| AMY, U/L | 52 (38 - 61) | 52.5 (37.25 - 63.25) | 62 (48.25 - 77.75) | 48 (38 - 57.5) | 0.001 |
| PA, mg/L | 192.97 ± 55.83 | 189.91 ± 64.47 | 191.75 ± 45.66 | 195.38 ± 54.12 | 0.868 |
| RBP, mg/L | 40.62 ± 16.09 | 43.13 ± 18.01 | 40.1 ± 13.98 | 39.25 ± 15.58 | 0.434 |
| ALT, U/L | 17.8 (12.5 - 30.8) | 20.05 (13.18 - 33.6) | 15.5 (11.35 - 25.5) | 17.8 (12.5 - 31.2) | 0.487 |
| GGT, U/L | 23 (14 - 39) | 23 (12.75 - 42.25) | 21 (15 - 39) | 24 (14 - 37.5) | 0.962 |
| AST, U/L | 21.3 (16.6 - 31.8) | 23.9 (18.3 - 39.83) | 21.3 (17.03 - 29.75) | 19.6 (15.5 - 27.9) | 0.077 |
| Hs-CRP, mg/L | 10.9 (2 - 26.4) | 8.4 (2.5 - 19.34) | 15.75 (4.88 - 23.53) | 9.5 (1.05 - 34.68) | 0.632 |
| APO-A, g/L | 0.93 (0.8 - 1.09) | 0.94 (0.8 - 1.1) | 1 (0.84 - 1.09) | 0.9 (0.8 - 1.08) | 0.350 |
| APO-B, g/L | 0.81 (0.7 - 1) | 0.8 (0.62 - 0.97) | 0.9 (0.76 - 1) | 0.81 (0.7 - 1.04) | 0.180 |
| HDL, mmol/L | 1.11 (0.9 - 1.38) | 1.11 (0.9 - 1.41) | 1.22 (1 - 1.4) | 1.1 (0.9 - 1.3) | 0.602 |
| HsCAR | 0.22 (0.04 - 0.56) | 0.18 (0.05 - 0.45) | 0.34 (0.1 - 0.52) | 0.2 (0.02 - 0.74) | 0.664 |
| HsCPAR | 0.06 (0.01 - 0.15) | 0.05 (0.01 - 0.12) | 0.09 (0.02 - 0.14) | 0.06 (0 - 0.2) | 0.705 |
| PNI | 478.25 (447.25 - 499.3) | 482.63 (443.75 - 502.36) | 474.9 (445.75 - 499.23) | 477.45 (447.28 - 497.1) | 0.892 |

Data are presented as median (interquartile range) or mean ± standard deviation. Continuous variables were analyzed by variance analysis or Kruskal‒Wallis test. A *P* value of less than 0.05 (two-tailed) was considered statistically significant.

**Abbreviations:** IL-6, interlukin-6; RDW-CV, red cell distribution width-coefficient of variation; NEU, neutrophil; MON, monocyte; BA, Basophils; EO, Eosinophils; MPV, mean platelet volume; MCHC, Mean corpuscular hemoglobin concentration; MCH, Mean corpuscular hemoglobin; LYM, lymphocyte; WBC, white blood cell; RBC, red blood cell; RDW-SD, red cell distribution width-standard deviation; MCV, mean corpuscular volume; PLT, platelet; PCT, Plateletcrit; HB, Hemoglobin; NLR, neutrophil-lymphocyte ratio; PLR, platelet-lymphocyte ratio; LMR, lymphocyte-monocyte ratio; dNLR, derived neutrophil-lymphocyte ratio; AFR, albumin-to-fibrinogen ratio; SⅡ, systemic immune-inflammation index; PT, prothrombin time; PTA, prothrombin activity; TT, thrombin time; INR, international normalized ratio; AT Ⅲ, Antithrombin Ⅲ; FDP, fibrinogen degradation product; FBG, fibrinogen; APTT, acivated partial thromboplastin time; HBDH, alpha-hydroxybutyric acid; LDH, lactate dehydrogenase; LDL, low-density lipoprotein cholesterol; UA, uric acid; CHOL, total cholesterol; TP, total protein; GLB, globulin; TG, triglyceride; ALB,albumin; ALP, alkaline phosphatase; CREA, creatinine; CK, creatine kinase; CKMB, Creatine Kinase Isoenzyme; LPA, Lipoprotein A; ADA, Adenosine Deaminase; AMY, amylase; PA, prealbumin; RBP, retinol binding protein; ALT, alanine aminotransferase; GGT, γ-glutamyltransferase; AST, aspartate aminotransferase; HsCRP, high sensitivity Creactive protein; APO, Apolipoprotein; HDL, high density liptein cholesterol; HsCAR, high sensitivity C-reactive protein-albumin ratio; HsCPAR, high sensitivity C-reactive protein-prealbumin ratio; PNI, prognostic nutritional index.
