# Supplementary material for: Comparison of the duration of viral RNA shedding and anti-SARS-CoV-2 spike IgG and IgM antibody titers in COVID-19 patients who were vaccinated with inactivated vaccines or not: a retrospective study
Source: BMC Infect Dis. 2022 Nov 9;22:831. doi: 10.1186/s12879-022-07808-2 (PMC9645737; doi:10.1186/s12879-022-07808-2)
Supplement: Supplementary file 4 — Additional file 4: Table S4. Proportions of abnormal level of IL-6, lymphocyte count, LDH, and D-dimmer in the three groups. [file 12879_2022_7808_MOESM4_ESM.docx]

**Additional file 4: Table S4. Proportion of Abnormal Level of IL-6, Lymphocyte Count, LDH, and D-dimmer in the Three Groups**

|  | **Total (n =147)** | **UV (n =46)** | **PV (n =28)** | **FV (n = 73)** | ***P*** |
| --- | --- | --- | --- | --- | --- |
| IL-6, >6.6pg/ml, No. (%) | 113 (76.9) | 36 (78.3) | 22 (78.6) | 55 (75.3) | 0.909 |
| RDW-CV, >14.8, No. (%) | 6 (4.1) | 3 (6.5) | 0 | 3 (4.1) |  |
| LYM, >3.2*10^9^/L, No. (%) | 79 (53.7) | 23 (50) | 17 (60.7) | 39 (53.4) | 0.667 |
| D-dimer, >0.55mg/L, No. (%) | 32 (21.8) | 9 (19.6) | 7 (25) | 16 (21.9) | 0.859 |
| HsCRP, >3mg/L, No. (%) | 107 (72.8) | 34 (73.9) | 23 (82.1) | 50 (68.5) | 0.378 |

Data are presented as the number (percentage). Categorical variables were compared using the chi-square (χ²) test or Fisher’s exact test. A *P* value of less than 0.05 (two-tailed) was considered statistically significant.

**Abbreviations:** IL-6, interlukin-6; RDW-CV, red cell distribution width-coefficient of variation; LYM, lymphocyte; HsCRP, high sensitivity C-reactive protein.
