# Supplementary material for: Comparison of the duration of viral RNA shedding and anti-SARS-CoV-2 spike IgG and IgM antibody titers in COVID-19 patients who were vaccinated with inactivated vaccines or not: a retrospective study
Source: BMC Infect Dis. 2022 Nov 9;22:831. doi: 10.1186/s12879-022-07808-2 (PMC9645737; doi:10.1186/s12879-022-07808-2)
Supplement: Supplementary file 5 — Additional file 5: Table S5. Ct value correlated with SARS-CoV-2 specific IgG and IgM Titers. [file 12879_2022_7808_MOESM5_ESM.docx]

**Additional file 5: Table S5. Ct Value Correlated with SARS-CoV-2 specific IgG and IgM Titers**

|  | **Ct value** | ***P*** |
| --- | --- | --- |
| SARS-CoV-2 specific IgG titer | r = 0.338 | 0 |
| SARS-CoV-2 specific IgM titer | r = 0.379 | 0 |

Correlation analysis was performed using the Spearman method. Spearman correlation coefficient was represented by the r value. A *P* value of less than 0.05 (two-tailed) was considered statistically significant.

**Abbreviations:** Ct, SARS-CoV-2 nucleocapsid gene cycle threshold (ct) value.
