# Supplementary material for: Comparison of the duration of viral RNA shedding and anti-SARS-CoV-2 spike IgG and IgM antibody titers in COVID-19 patients who were vaccinated with inactivated vaccines or not: a retrospective study
Source: BMC Infect Dis. 2022 Nov 9;22:831. doi: 10.1186/s12879-022-07808-2 (PMC9645737; doi:10.1186/s12879-022-07808-2)
Supplement: Supplementary file 6 — Additional file 6: Table S6. Laboratory tests of the three groups with comorbidities. [file 12879_2022_7808_MOESM6_ESM.docx]

**Additional file 6: Table S6. Laboratory Tests of the Three Groups with Comorbidities**

|  | **Total (n = 54)** | **UV (n = 22)** | **PV (n = 13)** | **FV (n = 19)** | ***P*** |
| --- | --- | --- | --- | --- | --- |
|  |  |  |  |  |  |
| IL-6, pg/ml | 16.35 (8.3 - 30.63) | 13.4 (8.28 - 29.9) | 13.2 (6.95 - 29.35) | 23.2 (11.2 - 31.7) | 0.609 |
| RDW-CV | 12.3 (11.9 - 12.6) | 12.35 (12.08 - 13.38) | 12.2 (11.65 - 12.5) | 12.3 (11.9 - 12.5) | 0.179 |
| NEU, ×10^9^/l | 3.21 (2.67 - 4.26) | 3.1 (2.24 - 4.15) | 3 (2.75 - 6.35) | 3.5 (2.8 - 4.2) | 0.322 |
| MON, ×10^9^/l | 0.5 (0.4 - 0.7） | 0.5 (0.37 - 0.72) | 0.46 (0.33 - 0.65) | 0.5 (0.4 - 0.69) | 0.659 |
| MPV, fL | 10.85 (10.58 - 11.5) | 10.9 (10.45 - 11.43) | 11.5 (10.75 - 12.3) | 10.8 (10.1 - 11.3) | 0.010 |
| MCHC, g/L | 342.5 (334.75 - 352) | 342.5 (331.5 - 352) | 345 (335 - 353) | 342 (334 - 354) | 0.670 |
| MCH, pg | 30.3 (29.1 - 31.1 | 29.85 (29 - 30.63) | 31.3 (30.2 - 32.5) | 30.3 (29.1 - 31) | 0.015 |
| LYM, ×10^9^/l | 1.05 (0.74 - 1.5) | 1.06 (0.69 - 1.48) | 0.86 (0.65 - 1.34) | 1.2 (0.98 - 1.7) | 0.139 |
| WBC, ×10^9^/l | 5.4 ± 1.62 | 4.98 ± 1.45 | 5.92 ± 2.26 | 5.52 ± 1.18 | 0.263 |
| RBC, ×10^12^/l | 4.46 ± 0.58 | 4.34 ± 0.59 | 4.3 ± 0.52 | 4.71 ± 0.54 | 0.066 |
| RDW-SD | 39.57 ± 4.65 | 37.7 ± 4.62 | 40.27 ± 4.81 | 41.26 ± 3.95 | 0.038 |
| MCV, fL | 88.1 (85.78 - 90.13) | 87.2 (83.88 - 89.15) | 90.2 (87.3 - 93.4) | 88.3 (85.7 - 89.8) | 0.039 |
| PLT, ×10^9^/l | 185 (136 - 216) | 153.5 (127.75 - 198.75) | 170 (109 - 205.5) | 207 (185 - 256) | 0.014 |
| PCT | 0.2 (0.16 - 0.21) | 0.2 (0.13 - 0.21) | 0.2 (0.12 - 0.21) | 0.2 (0.2 - 0.3) | 0.104 |
| HB, g/L | 133.72 ± 18.74 | 126.95 ± 21.11 | 133.69 ± 13.09 | 141.58 ± 16.71 | 0.042 |
| NLR | 3.1 (2.18 - 4.7) | 3.05 (2.25 - 3.53) | 4.2 (2.4 - 6.5) | 2.6 (2 - 3.9) | 0.087 |
| LMR | 1.9 (1.4 - 3.13) | 1.85 (1.4 - 2.7) | 1.8 (1.25 - 3.55) | 2.2 (1.4 - 3.6) | 0.664 |
| PLR | 173.5 (129.75 - 208.3) | 158.75 (103.5 - 198.2) | 178.3 (129.7 - 292.6) | 176.6 (139.8 - 212.2) | 0.795 |
| dNLR | 1.9 (1.5 - 2.63) | 1.9 (1.48 - 2.3) | 2.6 (1.7 - 4.15) | 1.8 (1.5 - 2.5) | 0.063 |
| SⅡ | 514.1 (366.2 - 878.48) | 426.9 (309.88 - 672.83) | 517.8 (389.85 - 1459.8) | 545.8 (448.7 - 690.2) | 0.349 |
| AFR | 13.05 (11.13 - 16.4) | 13.45 (11.73 - 16.83) | 12.8 (11.65 - 17.9) | 12.9 (8.9 - 14.6) | 0.186 |
| D-dimer, mg/L | 0.38 (0.2 - 0.63) | 0.39 (0.2 - 0.82) | 0.4 (0.3 - 0.95) | 0.22 (0.2 - 0.5) | 0.151 |
| PT, s | 11.75 ± 0.84 | 11.67 ± 0.83 | 11.35 ± 0.46 | 12.13 ± 0.92 | 0.027 |
| PTA | 92.09 ± 13.91 | 93.73 ± 15.12 | 98.45 ± 9.2 | 85.83 ± 13.15 | 0.029 |
| TT, s | 17.9 (17.4 - 18.8) | 17.95 (17.4 - 18.8) | 18 (17.35 - 18.8) | 17.7 (17.1 - 18.4) | 0.639 |
| INR | 1 (0.99 - 1.1) | 1 (0.97 - 1.1) | 1 (0.96 - 1) | 1.07 (1 - 1.1) | 0.035 |
| AT Ⅲ | 90.39 ± 11.95 | 88.45 ± 14.18 | 92.45 ± 9.57 | 91.24 ± 10.79 | 0.596 |
| FDP, ug/ml | 2.02 (1.63 - 2.59) | 2.21 (1.63 - 3.1) | 2.21 (2.1 - 3.73) | 1.8 (1.4 - 2.02) | 0.020 |
| FBG, g/L | 3.59 ± 0.98 | 3.36 ± 0.79 | 3.32 ± 0.82 | 4.02 ± 1.16 | 0.051 |
| APTT, s | 28.85 (26.35 - 30.93) | 29.3 (27.4 - 32.5) | 27 (25.6 - 30.6) | 28.5 (27.6 - 30.8) | 0.436 |
| HBDH, U/L | 155.5 (135 - 182.5) | 156.5 (146 - 183.25) | 165 (133 - 178.5) | 152 (128 - 191) | 0.692 |
| LDH, IU/L | 200.5 (180 - 238) | 205 (194.5 - 239.25) | 199 (176.5 - 239) | 197 (171 - 252) | 0.674 |
| LDL, mmol/L | 1.95 (1.4 - 2.56 0 | 1.59 (1.26 - 2.35) | 2.2 (1.68 - 2.76) | 2.1 (1.7 - 2.6) | 0.072 |
| UA, μmol/L | 4.85 (3.9 - 5.88) | 4.65 (3.85 - 6.4) | 5 (4.35 - 5.3) | 5.1 (3.6 - 6.4) | 0.921 |
| CHOL, mmol/L | 3.95 (3.19 - 4.29) | 3.4 (2.94 - 4.12) | 4.3 (3.7 - 4.92) | 3.9 (3.18 - 4.26) | 0.058 |
| TP, g/L | 74.15 (70 - 78.63) | 74.15 (71.15 - 79.45) | 75 (70.6 - 78.6) | 73.3 (68.5 - 78) | 0.845 |
| GLB, g/L | 27.69 ± 4.43 | 27.8 ± 4.4 | 28.24 ± 5.69 | 27.18 ± 3.63 | 0.800 |
| TG, mmol/L | 1.54 (1 - 2) | 1.15 (0.91 - 1.97) | 1.41 (1 - 2.78) | 1.79 (1.13 - 2) | 0.541 |
| ALB, g/L | 45.8 ± 4.7 | 45.92 ± 4.69 | 44.85 ± 5.48 | 46.31 ± 4.29 | 0.692 |
| A/G | 1.7 ± 0.34 | 1.69 ± 0.33 | 1.66 ± 0.41 | 1.73 ± 0.33 | 0.844 |
| ALP, U/L | 87 (74 - 100.75) | 84 (73 - 98.25) | 86 (72.5 - 106.5) | 89 (76 - 103) | 0.988 |
| CREA, μmol/L | 71 (64.75 - 92) | 70.5 (64.75 - 94.25) | 71 (65.5 - 102.5) | 77 (60 - 91) | 0.965 |
| CK, U/L | 94.5 (60 - 179.75) | 99 (65.25 - 241) | 63 (51.5 - 120) | 141 (68 - 201) | 0.153 |
| CKMB, U/L | 13.4 (10.15 - 15.55) | 13.45 (10.98 - 15.83) | 10.2 (8.5 - 14.7) | 13.7 (10.6 - 16.9) | 0.331 |
| LPA, mg/L | 65 (30.95 - 168.65) | 47.3 (26.15 - 114.5) | 167.5 (45.45 - 354.4) | 70 (24.6 - 158.3) | 0.107 |
| ADA, U/L | 14 (12 - 16.25) | 15 (13.75 - 17.25) | 13 (11.5 - 15.5) | 14 (12 - 16) | 0.195 |
| AMY, U/L | 48.5 (35.75 - 59.75) | 40 (32.75 - 56.25) | 59 (45.5 - 86) | 41 (37 - 58) | 0.020 |
| PA, mg/L | 192.14 ± 62.69 | 184.95 ± 73.38 | 186.6 ± 49.54 | 204.26 ± 58.42 | 0.585 |
| RBP, mg/L | 47.06 ± 17.49 | 47.38 ± 21.03 | 41.42 ± 13.83 | 50.56 ± 14.87 | 0.354 |
| ALT, U/L | 20.05 (14.38 - 33.13) | 22.85 (14.45 - 40.78) | 18.3 (13.35 - 31.9) | 18.9 (14.3 - 30.8) | 0.672 |
| GGT, U/L | 30 (15 - 59.25) | 23.5 (12 - 52.5) | 25 (15 - 60.5) | 33 (26 - 67) | 0.458 |
| AST, U/L | 24.7 (18.83 - 37.33) | 32.65 (20.93 - 44.65) | 21.5 (18.65 - 30.95) | 20 (18.1 - 27.4) | 0.038 |
| Hs-CRP, mg/L | 16.49 (4.68 - 33.81) | 14.7 (4.9 - 28.43) | 16.68 (3.4 - 30.36) | 28.8 (4 - 40.4) | 0.538 |
| APO-A, g/L | 0.93 ± 0.21 | 0.92 ± 0.21 | 0.98 ± 0.2 | 0.91 ± 0.22 | 0.632 |
| APO-B, g/L | 0.9 (0.7 - 1) | 0.73 (0.6 - 0.9) | 1 (0.9 - 1.05) | 0.9 (0.75 - 1.07) | 0.041 |
| HDL, mmol/L | 1 (0.82 - 1.3) | 0.9 (0.8 - 1.3) | 1.2 (0.85 - 1.4) | 0.9 (0.9 - 1.2) | 0.494 |
| HsCAR | 0.38 (0.09 - 0.74) | 0.33 (0.12 - 0.56) | 0.37 (0.07 - 0.7) | 0.58 (0.08 - 0.9) | 0.602 |
| HsCPAR | 0.1 (0.02 - 0.17) | 0.1 (0.03 - 0.14) | 0.09 (0.01 - 0.21) | 0.12 (0.02 - 0.26) | 0.989 |
| PNI | 463.86 ± 47.74 | 464.9 ± 47.48 | 453.46 ± 55.26 | 469.79 ± 43.91 | 0.640 |

Data are presented as median (interquartile range) or mean ± standard deviation. Continuous variables were analyzed by variance analysis or Kruskal‒Wallis test. A *P* value of less than 0.05 (two-tailed) was considered statistically significant.

**Abbreviations:** IL-6, interlukin-6; RDW-CV, red cell distribution width-coefficient of variation; NEU, neutrophil; MON, monocyte; BA, Basophils; EO, Eosinophils; MPV, mean platelet volume; MCHC, Mean corpuscular hemoglobin concentration; MCH, Mean corpuscular hemoglobin; LYM, lymphocyte; WBC, white blood cell; RBC, red blood cell; RDW-SD, red cell distribution width-standard deviation; MCV, mean corpuscular volume; PLT, platelet; PCT, Plateletcrit; HB, Hemoglobin; NLR, neutrophil-lymphocyte ratio; PLR, platelet-lymphocyte ratio; LMR, lymphocyte-monocyte ratio; dNLR, derived neutrophil-lymphocyte ratio; AFR, albumin-to-fibrinogen ratio; SⅡ, systemic immune-inflammation index; PT, prothrombin time; PTA, prothrombin activity; TT, thrombin time; INR, international normalized ratio; ATⅢ, Antithrombin Ⅲ; FDP, fibrinogen degradation product; FBG, fibrinogen; APTT, acivated partial thromboplastin time; HBDH, alpha-hydroxybutyric acid; LDH, lactate dehydrogenase; LDL, low-density lipoprotein cholesterol; UA, uric acid; CHOL, total cholesterol; TP, total protein; GLB, globulin; TG, triglyceride; ALB,albumin; ALP, alkaline phosphatase; CREA, creatinine; CK, creatine kinase; CKMB, Creatine Kinase Isoenzyme; LPA, Lipoprotein A; ADA, Adenosine Deaminase; AMY, amylase; PA, prealbumin; RBP, retinol binding protein; ALT, alanine aminotransferase; GGT, γ-glutamyltransferase; AST, aspartate aminotransferase; Hs-CRP, high sensitivity C-reactive protein; APO, Apolipoprotein; HDL, high density liptein cholesterol; HsCAR, high sensitivity C-reactive protein-albumin ratio; HsCPAR, high sensitivity C-reactive protein-prealbumin ratio; PNI, prognostic nutritional index.
