# Supplementary material for: Comparison of the duration of viral RNA shedding and anti-SARS-CoV-2 spike IgG and IgM antibody titers in COVID-19 patients who were vaccinated with inactivated vaccines or not: a retrospective study
Source: BMC Infect Dis. 2022 Nov 9;22:831. doi: 10.1186/s12879-022-07808-2 (PMC9645737; doi:10.1186/s12879-022-07808-2)
Supplement: Supplementary file 7 — Additional file 7: Table S7. Laboratory tests of the three groups with hypertension. [file 12879_2022_7808_MOESM7_ESM.docx]

**Supplementary Table 7. Laboratory Tests of the Three Groups with Hypertension**

|  | **Total (n = 40)** | **UV (n = 16)** | **PV (n = 10)** | **FV (n = 14)** | ***P*** |
| --- | --- | --- | --- | --- | --- |
|  |  |  |  |  |  |
| IL-6, pg/ml | 14.8 (8.05 - 28.7) | 12.55 (8.05 - 29.48) | 10.6 (4.93 - 22.95) | 23.6 (9.93 - 32.28) | 0.386 |
| RDW-CV | 12.3 (11.9 - 12.5) | 12.35 (12.13 - 13.73) | 12.25 (11.8 - 12.5) | 12.1 (11.78 - 12.4) | 0.125 |
| NEU, ×10^9^/l | 3.21 (2.54 - 4.45) | 2.95 (2.03 - 4.38) | 3.1 (2.78 - 7.18) | 3.7 (2.72 - 4.45) | 0.267 |
| MON, ×10^9^/l | 0.5 (0.39 - 0.62) | 0.48 (0.31 - 0.68) | 0.43 (0.34 - 0.53) | 0.55 (0.4 - 0.72) | 0.299 |
| MPV, fL | 11.12 ± 0.88 | 11.24 ± 0.96 | 11.39 ± 0.7 | 10.8 ± 0.85 | 0.215 |
| MCHC, g/L | 345 (337 - 353.75) | 343 (329.5 - 352.75) | 346.5 (335 - 352) | 346.5 (337.75 - 355.25) | 0.676 |
| MCH, pg | 30.3 (29.03 - 31.18) | 29.4 (27.88 - 30.75) | 31.1 (29.8 - 33.1) | 30.55 (29.05 - 31.33) | 0.061 |
| LYM, ×10^9^/l | 1.2 (0.71 - 1.51) | 1.21 (0.68 - 1.5) | 0.86 (0.68 - 1.35) | 1.25 (0.88 - 1.7) | 0.3 |
| WBC, ×10^9^/l | 5.39 (4.05 - 6.76) | 4.88 (3.19 - 6.54) | 5.3 (4.12 - 8.55) | 6.25 (4.45 - 6.72) | 0.244 |
| RBC, ×10^12^/l | 4.5 ± 0.57 | 4.45 ± 0.63 | 4.4 ± 0.56 | 4.62 ± 0.54 | 0.592 |
| RDW-SD | 39.58 ± 4.57 | 38.23 ± 5.03 | 39.99 ± 4.49 | 40.83 ± 3.94 | 0.291 |
| MCV, fL | 87.75 (84.95 - 89.75) | 86.15 (82.45 - 88.5) | 89.9 (87.3 - 92.9) | 88.15 (85.48 - 89.43) | 0.034 |
| PLT, ×10^9^/l | 189 (139 - 220.5) | 153.5 (135.25 - 196) | 183.5 (130.25 - 216) | 214 (187.25 - 253.5) | 0.03 |
| PCT | 0.2 (0.17 - 0.21) | 0.2 (0.15 - 0.21) | 0.2 (0.15 - 0.23) | 0.2 (0.2 - 0.3) | 0.31 |
| HB, g/L | 134.5 (122 - 154) | 127.5 (116.5 - 150.75) | 135.5 (125 - 144.75) | 139.5 (125.75 - 157) | 0.347 |
| NLR | 2.9 (2.23 - 4.2) | 2.75 (2.15 - 3.43) | 4 (2.55 - 8.3) | 2.6 (2.1 - 4.33) | 0.134 |
| LMR | 2.1 (1.4 - 3.35) | 1.95 (1.4 - 3.08) | 2.1 (1.33 - 3.9) | 2.25 (1.4 - 3.73) | 0.944 |
| PLR | 168.7 (121.5 - 217.3) | 150.7 (102.5 - 201.4) | 181.2 (129.75 - 336.4) | 187.9 (120.25 - 214.3) | 0.634 |
| dNLR | 1.9 (1.53 - 2.75) | 1.85 (1.43 - 2.23) | 2.5 (1.75 - 5.05) | 1.8 (1.5 - 2.8) | 0.138 |
| SⅡ | 506.75 (345.88 - 897.03) | 385.85 (297.43 - 641.55) | 712.05 (401.33 - 1719.28) | 570.8 (421.48 - 804.4) | 0.168 |
| AFR | 13.15 (11.53 - 17.55) | 13.8 (11.95 - 18.05) | 12.85 (12.03 - 18.33) | 13.25 (9.58 - 15.15) | 0.219 |
| D-dimer, mg/L | 0.4 (0.2 - 0.59) | 0.39 (0.2 - 0.75) | 0.48 (0.3 - 0.93) | 0.25 (0.2 - 0.49) | 0.293 |
| PT, s | 11.6 (11.2 - 12.3) | 11.6 (11.13 - 12.35) | 11.4 (10.95 - 11.73) | 11.85 (11.48 - 12.45) | 0.173 |
| PTA | 92.34 ± 13.78 | 93.77 ± 14.76 | 97.63 ± 10.26 | 86.92 ± 13.78 | 0.149 |
| TT, s | 18.05 ± 1.01 | 18.06 ± 0.95 | 17.93 ± 0.9 | 18.13 ± 1.19 | 0.897 |
| INR | 1 (0.99 - 1.1) | 1 (0.96 - 1.1) | 1 (0.97 - 1.01) | 1.04 (1 - 1.1) | 0.212 |
| ATⅢ | 90.75 ± 11.4 | 88.06 ± 12.78 | 92.67 ± 9.99 | 92.44 ± 10.84 | 0.489 |
| FDP, ug/ml | 2.02 (1.63 - 2.54) | 2.21 (1.68 - 2.9) | 2.21 (2.15 - 3.55) | 1.8 (1.4 - 2.01) | 0.024 |
| FBG, g/L | 3.52 ± 1.03 | 3.3 ± 0.89 | 3.29 ± 0.82 | 3.94 ± 1.23 | 0.178 |
| APTT, s | 29 (26.55 - 30.8) | 29.3 (27.75 - 31.88) | 26.6 (25.7 - 29.98) | 29.4 (28.05 - 30.83) | 0.307 |
| HBDH, U/L | 163.78 ± 31.52 | 163.06 ± 26.73 | 155.4 ± 26.89 | 170.57 ± 39.39 | 0.517 |
| LDH, IU/L | 202 (181 - 250) | 205 (195.25 - 230) | 191 (171.5 - 236.5) | 211.5 (175.5 - 271) | 0.566 |
| LDL, mmol/L | 1.88 ± 0.74 | 1.56 ± 0.68 | 2.11 ± 0.69 | 2.08 ± 0.73 | 0.071 |
| UA, μmol/L | 4.75 (4.08 - 6.23) | 4.6 (4.03 - 6.13) | 4.8 (3.98 - 5.75) | 5.1 (4.15 - 6.48) | 0.838 |
| CHOL, mmol/L | 3.7 (3.1 - 4.24) | 3.2 (2.59 - 4.11) | 4.18 (3.61 - 5.03) | 3.65 (3.16 - 4.21) | 0.064 |
| TP, g/L | 73.82 ± 6.21 | 73.83 ± 7.13 | 73.78 ± 6.43 | 73.85 ± 5.33 | 1 |
| GLB, g/L | 27.62 ± 4.74 | 27.2 ± 4.63 | 27.75 ± 6.28 | 28.01 ± 3.88 | 0.896 |
| TG, mmol/L | 1.66 (1.02 - 2.25) | 1.56 (0.92 - 2.63) | 1.65 (0.96 - 5.2) | 1.8 (1.18 - 2.02) | 0.807 |
| ALB, g/L | 46.2 ± 4.24 | 46.63 ± 4.51 | 46.03 ± 4.1 | 45.84 ± 4.29 | 0.875 |
| A/G | 1.73 ± 0.35 | 1.76 ± 0.33 | 1.75 ± 0.42 | 1.66 ± 0.32 | 0.725 |
| ALP, U/L | 87 (74.5 - 98.5) | 84 (74.5 - 93) | 94.5 (75.25 - 112.25) | 86.5 (70 - 97) | 0.482 |
| CREA, μmol/L | 70.5 (64 - 90.75) | 71 (64.75 - 107.75) | 69 (62.75 - 85.75) | 70 (57.25 - 92) | 0.618 |
| CK, U/L | 98.5 (60.75 - 200) | 98.5 (63.75 - 356.5) | 60.5 (47.5 - 99) | 144.5 (84.5 - 203) | 0.052 |
| CKMB, U/L | 13.5 (9.93 - 15.65) | 13.6 (10.23 - 16.08) | 11.55 (7.73 - 14.65) | 14 (10.2 - 18.48) | 0.309 |
| LPA, mg/L | 47.9 (22.73 - 165.2) | 36.65 (21.8 - 71.8) | 122.5 (51.08 - 515.58) | 51.45 (19.38 - 169.25) | 0.117 |
| ADA, U/L | 14 (12 - 16) | 15 (14 - 16) | 13 (11.75 - 14) | 15 (12 - 18.5) | 0.127 |
| AMY, U/L | 48.5 (35.25 - 63.5) | 40 (33 - 56.5) | 61 (40.5 - 87.75) | 46.5 (39 - 55.25) | 0.150 |
| PA, mg/L | 196.76 ± 60.71 | 192.91 ± 71.09 | 200.81 ± 45.59 | 198.27 ± 61.26 | 0.946 |
| RBP, mg/L | 46.9 ± 18.08 | 49.28 ± 22.65 | 42.39 ± 15.16 | 47.39 ± 14.39 | 0.646 |
| ALT, U/L | 25.4 (15.33 - 35.45) | 25.4 (14.43 - 40.1) | 20.45 (13.68 - 33.13) | 28.1 (15.85 - 31.4) | 0.680 |
| GGT, U/L | 31 (15 - 63) | 28 (12.25 - 59.5) | 36 (15 - 66.75) | 30 (19.75 - 67.25) | 0.757 |
| AST, U/L | 25.8 (19.08 - 38.9) | 35.05 (22.08 - 43.25) | 20.95 (17.9 - 29.48) | 24.45 (19 - 38) | 0.084 |
| Hs-CRP, mg/L | 16.2 (3.98 - 29.34) | 14.65 (5.49 - 28.93) | 12.1 (1.98 - 30.5) | 23.2 (3.03 - 42.93) | 0.687 |
| APO-A, g/L | 0.92 ± 0.2 | 0.89 ± 0.17 | 0.95 ± 0.21 | 0.93 ± 0.22 | 0.734 |
| APO-B, g/L | 0.83 ± 0.22 | 0.73 ± 0.19 | 0.95 ± 0.21 | 0.86 ± 0.22 | 0.04 |
| HDL, mmol/L | 1.03 ± 0.3 | 0.95 ± 0.26 | 1.07 ± 0.37 | 1.1 ± 0.31 | 0.365 |
| HsCAR | 0.33 (0.08 - 0.63) | 0.3 (0.12 - 0.57) | 0.25 (0.04 - 0.66) | 0.49 (0.06 - 1) | 0.9 |
| HsCPAR | 0.09 (0.01 - 0.15) | 0.1 (0.03 - 0.14) | 0.06 (0.01 - 0.18) | 0.11 (0.01 - 0.28) | 0.939 |
| PNI | 467.99 ± 43.44 | 471.99 ± 46.23 | 465.35 ± 41.06 | 465.3 ± 44.65 | 0.898 |

Data are presented as median (interquartile range) or mean ± standard deviation. Continuous variables were analyzed by variance analysis or Kruskal‒Wallis test. A *P* value of less than 0.05 (two-tailed) was considered statistically significant.

**Abbreviations:** IL-6, interlukin-6; RDW-CV, red cell distribution width-coefficient of variation; NEU, neutrophil; MON, monocyte; BA, Basophils; EO, Eosinophils; MPV, mean platelet volume; MCHC, Mean corpuscular hemoglobin concentration; MCH, Mean corpuscular hemoglobin; LYM, lymphocyte; WBC, white blood cell; RBC, red blood cell; RDW-SD, red cell distribution width-standard deviation; MCV, mean corpuscular volume; PLT, platelet; PCT, Plateletcrit; HB, Hemoglobin; NLR, neutrophil-lymphocyte ratio; PLR, platelet-lymphocyte ratio; LMR, lymphocyte-monocyte ratio; dNLR, derived neutrophil-lymphocyte ratio; AFR, albumin-to-fibrinogen ratio; SⅡ, systemic immune-inflammation index; PT, prothrombin time; PTA, prothrombin activity; TT, thrombin time; INR, international normalized ratio; AT Ⅲ, Antithrombin Ⅲ; FDP, fibrinogen degradation product; FBG, fibrinogen; APTT, acivated partial thromboplastin time; HBDH, alpha-hydroxybutyric acid; LDH, lactate dehydrogenase; LDL, low-density lipoprotein cholesterol; UA, uric acid; CHOL, total cholesterol; TP, total protein; GLB, globulin; TG, triglyceride; ALB, albumin; ALP, alkaline phosphatase; CREA, creatinine; CK, creatine kinase; CKMB, Creatine Kinase Isoenzyme; LPA, Lipoprotein A; ADA, Adenosine Deaminase; AMY, amylase; PA, prealbumin; RBP, retinol binding protein; ALT, alanine aminotransferase; GGT, γ-glutamyltransferase; AST, aspartate aminotransferase; HsCRP, high sensitivity Creactive protein; APO, Apolipoprotein; HDL, high density liptein cholesterol; HsCAR, high sensitivity C-reactive protein-albumin ratio; HsCPAR, high sensitivity C-reactive protein-prealbumin ratio; PNI, prognostic nutritional index.
